# 

**Published:** 1886-05

**Authors:** 


Page.

ORIGINAL COMMUNICATIONS:

On the Origin and Development of the Bacillus Tuberculosis in the Human Lung, Liver, Spleen, etc. H. D. Schmidt ---- 394

Modifications in the Methods of Treating Transverse Fractures of the Patella. G. A. Staples ---- 438

EDITORIAL:

Physicians' Protective Association ---- 460

Ipsis Consuescere Rebus ---- 462

SOCIETY REPORTS:

CHICAGO MEDICAL SOCIETY: *Stated Meeting March 1st.*

Digestion and Dyspepsia. P. C. Jensen ---- 463

Two Unique Cases of Vesico-Vaginal Fistula. J. H. Etheridge ---- 464

Ruptured Ovarian Cyst. H. P. Newman ---- 466

Degenerated Kidney. J. Frank ---- 473

Lanolin. R. Tilley ---- 474

*Stated Meeting March 15th.*

Two cases of Mitral Stenosis. R. H. Babcock ---- 475

Unique Case of Vesico-Vaginal Fistula. E. C. Dudley ---- 476

Removal of the Entire Lower Jaw Through the Mouth. W. L. Axford ---- 479

Symblepharon of the Lower Lid. A. P. Gilmore ---- 480

Encapsulated Sarcoma of the Thigh. C. T. Parkes ---- 482

BOOK REVIEWS:

The Management of Labour, etc. H. G. Landis ---- 483

Theory and Practice of Obstetrics. P. Careoux and S. Tarnier ---- 485

The Essentials of Histology. E. A. Schafer ---- 486

